# Regional Cooperative Pollution Control and Residents’ Health Expenditures: Empirical Evidence from China

**Published:** 2019-06

**Authors:** Yuhui DAI, Zhidong TAN, Jianhua TAN, Lina YAN

**Affiliations:** 1. Accounting School, Zhongnan University of Economics and Law, Wuhan, China; 2. Dongfang College, Zhejiang University of Finance and Economics, Haining, China

**Keywords:** Air pollution, Residents’ health expenditures, Regional cooperative pollution control

## Abstract

**Background:**

Environmental pollution seriously damages not only the ecosystem but also the well-being of residents. There is an association between air pollution and residential health expenditures. The aim of this study was to identify the association between pollution control and residents’ health expenditures.

**Methods:**

Using the pilot project of the regional cooperative pollution control in 28 cities required by the Chinese government as the quasi-natural experiment setting and the panel data of residents’ health expenditures in China from 2014 to 2017, the impact of pollution control on residents’ health expenditures was systematically explored through difference-in-difference approach.

**Results:**

Air quality of the 28 cities that adopted the regional cooperative pollution control policy improves significantly, thus indicating that this policy effectively solves the self-interest issue of local governments in pollution control, and residents’ health expenditures consequently decrease.

**Conclusion:**

This study enriches the paradigm that analyzes the relationship between air pollution and residents’ health expenditures and reveals the mechanism through which the regional cooperative pollution control reduces residents’ health expenditures.

## Introduction

In recent years, the air pollution that results from the extensive emission of exhaust makes China a polluted country. Air pollution significantly hinders the improvement of human capital. The increase in health expenditures decreases the life quality and happiness of residents. Thus, effectively identifying the impact of air pollution on residents’ health expenditures is of important theoretical and practical significance to controlling air pollution and improving residents’ health conditions.

According to the existing literatures, environmental pollution dramatically increases residents’ health expenditures (1–4). However, the existing literatures have two shortcomings. First, many current studies using SO_2_ as the proxy variable for environmental quality, which may produce certain errors. In 2006, the “China’s 11th Five-Year Plan” set the reduction of major SO_2_ by 10% as an obligatory performance indicator (5). Given the “strategic acts” of political officials to control SO_2_ under the promotion pressure, a notable decrease in SO_2_ emissions but poor improvement in air quality is observed. Second, missing variables are an issue. The expenditure behaviors of residents may be affected by multiple aspects. Several factors, such as the regional cultural differences and expenditure habits of residents, are difficult to measure.

In February 2017, the Ministry of Ecology and Environment (MEE) of China formulated the “Work Program for the Prevention and Control of Air Pollution in Beijing-Tianjin-Hebei Regions and their Surrounding Areas in 2017.” As stipulated, 28 regions (2 municipalities directly under the central government and 26 cities) must complete the air control tasks assigned to them within a specified time frame. The MEE continuously ranked, evaluated, and supervised the air quality improvement in the 28 cities. The suddenly intensified efforts of environmental governance in the 28 cities of North China provide an ideal example of difference-in-difference (DID) analysis in observing the effect of policies. This DID model can effectively overcome possible deviations in using proxy variables and alleviating the issue of missing variables.

This study aimed to answer two major questions. (a) Are any improvements present in air pollution after the regional cooperative pollution control? (b) Does improving air pollution affect the health expenditures of residents? Therefore, with the regional cooperative pollution control required by the MEE in China as the quasi-experiment, this study analyzes the impact of air quality improvement on residents’ health expenditures on the basis of the panel data of residents’ health expenditures through the DID method. The conclusions are expected to serve as a reference for the government in formulating environmental policies.

### Literature Review and Hypothesis Interaction between regional cooperative pollution control and air pollution improvement

How to balance economic development and environment protection, resource saving (6) and power efficiency improvement (7) is an important issue to be solved in China. In current environmental governance system, the Central and local governments have relatively different attitudes toward environmental policies. Given that policies are mainly formulated by the central government and implemented by local governments, a “policymaker–executor” relationship exists between the two bodies. The absence of supervision between the central and local governments lead to deviations in the policy implementation by local governments (8). Fiscal decentralization gave birth to “local development-oriented governments,” which created a politically lawful government model that prioritized economic development and growth (9). Local officials have a de facto initiative in implementing environmental policies (10). Under the promotion system that considers GDP as the main assessment index of officials, promotion largely depends on the growth rate of GDP (11–13). Therefore, local officials are not given sufficient incentives to improve the environmental quality within their jurisdictions (14, 15).

Air pollution control is susceptible to externalities, which further weakens the enthusiasm of local governments in environmental governance. The high infiltration and inseparability of environmental pollution exceed traditional administrative regions (16, 17). When local governments invest resources to control pollution, they perform well to the environmental quality of the local and surrounding areas, thus implying the existence of positive externalities in environmental governance. Similarly, local pollution spreads to surrounding areas, which in turn imply negative externalities in environmental governance (18, 19). Cross-regional environmental governance exceeds the jurisdiction of a local government.

After implementing the regional cooperative pollution control, the MEE has continuously ranked, assessed, and supervised the air quality improvement in the 28 cities. Moreover, this government agency takes the assessment and ranking results as an important comprehensive assessment of leader groups and cadres. That is, the policies put pressure on the local governments and officials to act on environmental governance. To comply with the long-term policies, the main polluters have increased their investment in environmental treatment, which consequently helps improve air quality. On this basis, the following hypothesis is introduced:

Hypothesis 1: The 28 cities that participate in the regional cooperative pollution control embrace remarkable improvement in air quality in the long window period.

### Interaction between the improvement of air pollution and residents’ health expenditures

Air pollution has affected urban residents negatively, for instance, increase the chances of contracting influenza, asthma, and respiratory etc. (1), thereby increasing the medical expenditures of residents. Furthermore, residents must pay high for protection and cleaning supplies to protect against air pollution and haze because long-term exposure to air pollution will significantly increase death rate and health expenditures. Existing literatures on environmental pollution and health expenditures has mainly focused on environmental pollution, residents’ income, and health and medical expenditures (2, 3). It is generally concluded that the total toxic pollutant emissions and the per capita urban environmental expenditures were significantly correlated with health expenditures (20, 21).

In summary, the deterioration of environmental quality increases residents’ health expenditures. Correspondingly, residents’ health expenditures are reduced relatively in regions with improved environmental quality in comparison with those in regions with poor quality. On this basis, the following hypothesis is introduced:

Hypothesis 2: The proportion of strong ties (proportion of relatives and friends) in villagers’ social networks positively influences their therapeutic participation in self-organization.

## Materials and Methods

### Sample and Data

This study used the 28 cities that implement the regional cooperative pollution control required by the Chinese government as the experimental group and the rest of the cities within the provinces as the control group (Table 1) to avoid the interference of unobservable provincial characteristics considerable.

**Table 1:** Pilot project cities in China

***Experimental group***	***Control group***
Beijing; Tianjin; Shijiazhuang, Tangshan, Baoding, Langfang, Cangzhou, Hengshui, Handan and Xingtai in Hebei Province; Taiyuan, Yangquan, Changzhi and Jincheng in Shanxi Province; Jinan, Zibo, Liaocheng, Dezhou, Binzhou, Jining and Heze in Shandong Province; and Zhengzhou, Xinxiang, Hebi, Anyang, Jiaozuo, Puyang and Kaifeng in Henan Province.	Qinhuangdao, Zhangjiakou and Chengde in Hebei Province; Datong, Shuozhou, Jinzhong, Yuncheng, Xinzhou, Linfen and Lvliang in Shanxi Province; Qingdao, Zaozhuang, Dongying, Yantai, Weifang, Tai’an, Weihai, Rizhao, Laiwu and Linyi in Shandong Province; and Luoyang, Xuchang, Pingdingshan, Luohe, Nanyang, Sanmenxia, Zhumadian, Shangqiu, Xinyang and Zhoukou in Henan Province.

Data on health expenditures, disposable income, education, and death rate are downloaded from the website of the National Bureau of Statistics and the China City Statistical Yearbook. Urban air quality data are obtained from the MEE data center. Data on the emissions of urban pollutants and characteristics are also acquired from the *China City Statistical Yearbook*. For the sake of robustness, the continuous variables are winsorized at 1% and 99% quintiles in the regression, and Stata 14 software is used to process the data.

### Research Variables

#### 1) Dependent variable

Residents’ health expenditures represent the proportion of health expenditures to the total consumption expenditures. By analyzing this variable, this study can understand the trend of health expenditures and the attention accorded by residents on health.

#### 2) Independent variable

Regional cooperative pollution control is a dummy variable for measuring the effect before and after implementing the policy. The variable specifically takes the value of “0” for the 28 cities in 2014–2016 before the pilot project, “1” in 2017 after the pilot project, and “0” for the corresponding cities in the control group in 2014–2017.

#### 3) Control variables

The characteristics of residents will affect the policy effects on residents after the pilot project. In this study, disposable income, education, and death rate are used to measure the characteristics of residents. The variables are defined in Table 2.

**Table 2:** Definition of variables used in this study

***Variable***	***Symbol***	***Name***	***Declaration***
Dependent variable	He	Residents’ health expenditures	(health expenditures/consumption expenditures) ^*^100
Independent variable	Joint	The regional cooperative pollution control	0 before the pilot project and 1 thereafter, and 0 for the control group.
Residents’ characteristics	Y	Disposable income	Logarithmic per capita disposable income
	Edu	Education	Ratio of teachers to students in colleges and universities
	Death	Death rate	Death rate

### Model Design

Based on the theoretical analysis and defined variables and referring to the research of Duchin *et al.* (22), the multiple time points DID model is constructed as follows:

[I] $$He_{i,t}=\alpha +\sigma +\delta +\beta *Joint_{i,t}+\eta *X+\varepsilon$$

This study applies Model (I) to analyze the panel data of residents’ health expenditures from 2014 to 2017. Furthermore, the city- and year-fixed effects are controlled to avoid the possible effects of individual heterogeneity and time variation. The dependent variable is residents’ health expenditures (*He*), which indicate the proportion of residents’ health expenditures to the total consumption expenditures. *α* is the intercept term of the model. *σ* is the year effect; *δ* is the individuality effect of a city. *X* is a group of control variables, which controls the disposable income, health status, and education of residents; and *ε* is a random disturbance term.

### Descriptive statistics

In Table 3, the mean value of residents’ health expenditures is 7.231. The proportion of residents’ health expenditures to the total consumption expenditures is increasing. The annual gross growth of residents’ health expenditures in China has exceeded 16%, and air pollution is an important reason for the increase.

**Table 3:** Characteristics of the study samples (2014–2017)

***Variables***	***Sample***	***Mean value***	***Standard*** ***deviation***	***Minimum*** ***value***	***50*** ***percentile***	***75*** ***percentile***	***Maximum*** ***value***
Residents’ health expenditures	232	7.231	0.287	4.112	7.623	10.236	12.314
The regional cooperative pollution control	232	0.187	0.381	0	0	0	1
Disposable income	232	9.047	1.345	7.939	9.354	10.021	10.454
Education	232	0.117	0.311	0.071	0.103	0.177	0.243
Death rate	232	5.964	0.677	4.213	5.994	6.423	7.288

## Results

### The air quality has improved significantly in the 28 cities

After implementing the regional cooperative pollution control, was there only a short-term governance effect, such as “APEC Blue” and “Parade Blue,” in the 28 cities? This study used the DID model to test the changes in the indicators related to air pollution in the 28 cities during the two-year window period before and after implementing the policy.

[II] $$AQI_{i,t}\mathrm{etc}.=\alpha +\sigma +\delta +\beta_{1}*\mathrm{Join}t_{i,t}+\eta *X+\varepsilon$$

The dependent variables are AQI index, PM2.5, PM10, SO2, NO2, CO, O3, and other air quality indicators, and the air quality data are from the MEE data center. Joint takes the value of 0 for the 28 cities from April 2016 to March 2017, 1 for April 2017 to March 2018, and 0 for the control group in the window period of 2 years before and after the event.

*a* is the intercept term of the model; *σ* is the year effect; *Φ* is the month effect; *δ* is the individuality effect of a city; and *X* is a group of control variables, which mainly includes the highest temperature (*Temp_H*), the lowest temperature (*Temp_L*), whether rain or snow occurs (*Snow/Rain*), and wind power (*Wind*), to control the impact of weather on air quality. Weekend and holiday are also controlled to exclude the impact of seasons and people’s working hours on air quality. *ε* is a random disturbance term. In Table 4, a comprehensive index of air quality, that is, the *AQI* index was significantly reduced at the 1% level after implementing the regional cooperative pollution control. In terms of sub-indexes, PM2.5, PM10, SO_2_, NO_2_, and CO was significantly reduced at the 1% level, while O_3_ significantly increased at the 1% level. O3 was mainly converted from the NO_X_ emitted by automobiles; therefore, enterprises are not the main source of NO_X_.

**Table 4:** Test of changes of air quality

***Variables***	***(1)*** ***AQIindex***	***(2)*** ***PM2.5***	***(3)*** ***PM10***	***(4)*** ***SO****_2_*	***(5)*** ***NO****_2_*	***(6)*** ***CO***	***(7)*** ***O****_3_*
Joint	−13.421*** (−11.52)	−11.895*** (−11.66)	−13.554*** (−8.83)	−5.530*** (−9.71)	−3.584*** (−10.75)	−0.276*** (−18.13)	6.181*** (11.53)
Wind	−4.853*** (−9.22)	−6.984*** (−15.16)	−4.459*** (−6.44)	−4.460*** (−17.34)	−5.500*** (−36.52)	−0.133*** (−19.39)	3.691*** (15.25)
Temp_H	2.218*** (18.66)	1.208*** (11.62)	3.308*** (21.14)	1.377*** (23.71)	1.045*** (30.72)	0.010*** (6.51)	1.927*** (35.26)
Temp_L	0.970*** (7.11)	1.340*** (11.21)	0.381** (2.12)	−1.144*** (−17.13)	−0.378*** (−9.68)	0.015*** (8.65)	0.145** (2.31)
Snow/Rain	0.799 (1.05)	3.792*** (5.70)	0.111 (0.11)	−1.488*** (−4.00)	−0.173 (−0.80)	0.048*** (4.80)	−5.128*** (−14.67)
Weekend	2.420*** (3.79)	1.561*** (2.79)	3.961*** (4.71)	1.241*** (3.97)	0.211 (1.15)	0.003 (0.34)	0.127 (0.43)
Holiday	−11.422*** (−7.58)	−9.104*** (−6.90)	−15.434*** (−7.77)	−0.732 (−0.99)	−8.106*** (−18.79)	−0.128*** (−6.52)	10.374*** (14.96)
Constant	162.163*** (52.01)	138.715*** (50.83)	173.508*** (42.26)	56.961*** (37.37)	59.346*** (66.51)	2.527*** (62.14)	7.948*** (5.54)
Year FE	Yes	yes	yes	yes	yes	yes	yes
Month FE	Yes	yes	yes	yes	yes	yes	yes
Code FE	Yes	yes	yes	yes	yes	yes	yes
Observations	25,252	25,252	25,252	25,252	25,252	25,252	25,252
R-squared	0.356	0.391	0.354	0.509	0.568	0.458	0.690
r2_a	0.354	0.389	0.352	0.508	0.566	0.456	0.689
F	190.7	221.3	189.2	357.8	452.6	291.2	766.5

*Note: t-statistics are in parentheses,* ***, ** *and* * *indicate significance at the 1%, 5% and 10% levels respectively.*

The NO_X_ in automobile exhaust would produce O3 under ultraviolet radiation, that is, “secondary photochemistry reaction”. After the cross-regional cooperative pollution control, PM2.5 and PM10 significantly declined. However, with increased visibility in air, O_3_ pollution was further aggravated. In general, the regional cooperative pollution control had a long-term effect on environmental governance, and a significant improvement in air pollution was observed during the long window period.

### The health expenditures have declined significantly in the 28 cities

In Table 5, residents’ health expenditures decreased at the 5% level, thereby indicating that residents’ health expenditures decreased after implementing the regional cooperative pollution control. Among the control variables, disposable income (*Y*) had no significant impact on residents’ health expenditures. Death rate (*Death*) significantly increased residents’ health expenditures at the 5% level, while education (*Edu*) significantly decreased the expenditures. In Table 5, the 28 cities that participated in the regional cooperative pollution control exhibited a significant decline in residents’ health expenditures in comparison with other cities in the same provinces. By controlling *Y*, *Death*, and *Edu*, the reduction in health expenditures was likely due to the air quality improvement after the regional cooperative pollution control.

**Table 5:** Test of changes in residents’ health expenditures

***Variables***	***(1)*** ***He***	***(2)*** ***He***
Joint	−0.039** (−2.52)	−0.031** (−2.06)
Y		−0.000 (−1.01)
Death		0.003** (−2.16)
Edu		−0.221** (2.22)
Constant	0.003*** (3.01)	0.002*** (2.83)
Year FE	Yes	yes
City FE	Yes	yes
Observations	232	232
R-squared	0.356	0.391

*Note: t-statistics are in parentheses,* ***, ** *and* * *indicate significance at the 1%, 5% and 10% levels respectively*

### Dynamic test of residents’ health expenditures

To observe the dynamic changes in policy effects after the regional cooperative pollution control, this study constructed the following dynamic model (23):

[III] $$He_{i,t}=\alpha +\sigma +\beta_{1}*\mathrm{Before}3+\beta_{2}*\mathrm{Before}2+\beta_{3}*\mathrm{Before}1+\beta_{4}*\mathrm{Current}+\eta *X+\varepsilon$$

Table 6 displays no significant difference in urban residents’ health expenditures between the experimental and control groups in the first 3 years before the regional cooperative pollution control, thus satisfying the parallel trend hypothesis. In the year of the regional cooperative pollution control, urban residents’ health expenditures of the experimental and control groups decreased significantly at the 5% level. The regression results showed that residents’ health expenditures had dropped remarkably after the regional cooperative pollution control.

**Table 6:** Dynamic test of residents’ health expenditures

***Variables***	***(1) He***	***(2) He***
Before_3_	0.085 (0.26)	0.068 (0.22)
Before_2_	0.010 (0.90)	0.009 (0.77)
Before_1_	−0.015 (−0.35)	−0.009 (−0.10)
Current	−0.044*** (−2.61)	−0.036** (−2.46)
Constant	0.003** (2.02)	0.002** (1.97)
Control		yes
Year FE	Yes	yes
City FE	Yes	yes
R-squared	0.223	0.281

Note: t-statistics are in parentheses, ***, ** and * indicate significance at the 1%, 5% and 10% levels respectively

### Robustness test

This study explored the impact of regional cooperative pollution control on residents’ health expenditures. The estimation bias in regression could be eliminated only when the pilot cities are selected randomly. To overcome the sample selection bias, this study adopted the two-stage regression (24). The first stage was the Probit pilot city selection model, through which the probability of a city becoming a pilot city was estimated, and the inverse Mills ratio *Imr* was calculated; *Imr* was added as a control variable in Model (I) in the second stage. The Probit model is constructed as follows:

[IV] $$\mathrm{Tes}t_{i,t}=\alpha +\eta *X+\varepsilon$$

Test marks whether the city is a pilot city. If *Test* equals 1, then the city belongs to the experimental group (28 cities). If *Test* equals 0, then the city belongs to the control group. *α* is a model intercept term, *X* stands for the factors that affect the probability of becoming a pilot city, and *ε* is a random disturbance term. This study measured *X* through two dimensions, namely, the intensities of urban pollution emissions and economic characteristics. The former is measured by the SO_2_ and dust discharges, whereas the latter is measured by the proportion of secondary industries (*Second*) and the GDP (*LnGDP*).

The results from Columns (2)–(3) in Table 7 showed that the direction and significance of the coefficients of the Joint variables are consistent with those presented in Table 5, thereby verifying the robustness of the principal regression.

**Table 7:** Robustness test: Heckman’s two-stage regression

***Stage I Regression***		***Stage II Regression***		
***Variables***	***(1)*** ***test***	***Variables***	***(2)*** ***He***	***(3)*** ***He***
Second	−0.006 (−0.69)	Imr	0.104 (0.35)	0.077 (0.20)
lnGDP	0.640*** (3.97)	Joint	−0.037** (−2.33)	−0.029** (−2.00)
SO_2_-d	42.262 (1.02)	Y		−0.000 (−1.00)
Dust-d	−23.778 (−0.74)	Death		0.003** (−2.08)
		Edu		−0.217** (2.05)
Constant	−11.202*** (−3.80)	Constant	0.003*** (2.89)	0.002*** (2.75)
Year FE	yes	Year FE	yes	Yes
		City FE	yes	Yes
Observations	230	Observations	232	232
		R-squared	0.352	0.387

*Note: t-statistics are in parentheses,* ***, ** *and* * *indicate significance at the 1%, 5% and 10% levels respectively*

## Discussion

On the basis of the above mentioned tests and analyses, all research hypotheses are confirmed. In cities where the regional cooperative pollution control was implemented, air pollution has improved, and residents’ health expenditures have reduced. First, Table 4 indicates that the air quality has improved significantly in the 28 cities after implementing the regional cooperative pollution control*.*

Short-term regional cooperative pollution controls, such as “APEC Blue” and “Parade Blue,” are short-lived because they were promoted by short-term but strong intervention measures, such as shutting down polluters and traffic restriction, which is consistent with the results of previous studies (18, 25–27).

For a favorable and benign environment, institutionalized regional cooperation in pollution control is vital, which extends the results of current literatures (28–31).

Second, the present study further explores the impact of air quality improvement on residents’ health expenditures on the basis of the effectiveness of the regional cooperative pollution control. Table 5 summarizes that improving air pollution has effectively reduced residents’ health expenditures. In existing literatures, PM10 or SO_2_ was used as the proxy variable of air pollution (2, 3, 21, 32). To overcome the innate shortcomings of proxy variables, the present study constructs a DID model on the basis of the foregoing quasi-natural event of the regional cooperative pollution control.

Third, previous studies have generally controlled residents’ disposable income level, health status, education, infrastructure construction, and investment in environmental governance (2, 21), however, these studies still do not solve the omitted variable issue. Therefore, using the DID model and with adjacent cities in North China sharing similar regional cultural traditions and residents’ expenditure habits included in the experimental and control groups, this study alleviates the possible effect of missing variables.

## Conclusion

The regional cooperative pollution control led by the central government is an effective measure and reduces “free riding” and regional protectionism in environmental governance successfully. Under the guidance and continuous promotion of the central government, local governments face increasing pressure to improve the environment. All the 28 cities exhibited considerable improvements in terms of pollution, and sustainable and strong environmental regulations can solve the dilemma of air pollution control effectively. The 28 cities where the authorities cooperate in pollution control continuously have experienced improve environmental quality and reduce residents’ health expenditures, which are conducive to improving people’s life quality. This study effectively identifies the relationship between air pollution and residents’ health expenditures. In addition, a strong environmental regulation can improve the environment, minimize residents’ exposure to environmental pollution, and reduce the health expenditures caused by air pollution.

## Ethical considerations

Ethical issues (Including plagiarism, Informed Consent, misconduct, data fabrication and/or falsification, double publication and/or submission, redundancy, etc.) have been completely observed by the authors.
